# Comparison of Monkeypox Virus Clade Kinetics and Pathology within the Prairie Dog Animal Model Using a Serial Sacrifice Study Design

**DOI:** 10.1155/2015/965710

**Published:** 2015-08-24

**Authors:** Christina L. Hutson, Darin S. Carroll, Nadia Gallardo-Romero, Clifton Drew, Sherif R. Zaki, Tamas Nagy, Christine Hughes, Victoria A. Olson, Jeanine Sanders, Nishi Patel, Scott K. Smith, M. Shannon Keckler, Kevin Karem, Inger K. Damon

**Affiliations:** ^1^Centers for Disease Control and Prevention, Poxvirus and Rabies Branch, Atlanta, GA 30333, USA; ^2^Department of Pathology, College of Veterinary Medicine, The University of Georgia, Athens, GA 30602, USA

## Abstract

*Monkeypox virus* (MPXV) infection of the prairie dog is valuable to studying systemic orthopoxvirus disease. To further characterize differences in MPXV clade pathogenesis, groups of prairie dogs were intranasally infected (8 × 10^3^ p.f.u.) with Congo Basin (CB) or West African (WA) MPXV, and 28 tissues were harvested on days 2, 4, 6, 9, 12, 17, and 24 postinfection. Samples were evaluated for the presence of virus and gross and microscopic lesions. Virus was recovered from nasal mucosa, oropharyngeal lymph nodes, and spleen earlier in CB challenged animals (day 4) than WA challenged animals (day 6). For both groups, primary viremia (indicated by viral DNA) was seen on days 6–9 through day 17. CB MPXV spread more rapidly, accumulated to greater levels, and caused greater morbidity in animals compared to WA MPXV. Histopathology and immunohistochemistry (IHC) findings, however, were similar. Two animals that succumbed to disease demonstrated abundant viral antigen in all organs tested, except for brain. Dual-IHC staining of select liver and spleen sections showed that apoptotic cells (identified by TUNEL) tended to colocalize with poxvirus antigen. Interestingly splenocytes were labelled positive for apoptosis more often than hepatocytes in both MPXV groups. These findings allow for further characterization of differences between MPXV clade pathogenesis, including identifying sites that are important during early viral replication and cellular response to viral infection.

## 1. Introduction

*Importance*. Since smallpox eradication,* Monkeypox virus* (MPXV) has become the most important human health threat within the* Orthopoxvirus* genus. Concern over the potential of the virus to move outside of its natural range, as well as the increasing population of unvaccinated people that are now susceptible to MPXV (due to cessation of smallpox vaccination), makes it important to have numerous well characterized animal models. Previous work demonstrated that the prairie dog MPXV model mimics human disease more closely than previous models, including the development of skin rash. Thus, utilizing this animal model, therapeutics can be tested at time of rash onset. Through the current study we have described the viral spread and pathology of the two MPXV clades within the prairie dog animal model. This model will continue to be important in testing novel therapeutics and next generation vaccines and thus the current study will be invaluable in evaluating future efficacy studies.

*Orthopoxvirus* members of the family* Poxviridae* include important current, or eradicated, human pathogens such as* Monkeypox virus* (MPXV) and* variola virus* (VARV, the causative agent of smallpox). These viruses are closely related and disease progression and presentation during human infections are clinically similar with the exception of lymphadenopathy associated with human MPXV infections. Smallpox was solely a human pathogen, and an intense international campaign using surveillance, containment, and vaccination led to eradication of disease. However MPXV is a zoonosis and remains endemic to the rain forests of Central and Western Africa, with reports of sporadic human outbreaks and areas of prevalent disease. In the era after eradication of smallpox and with the subsequent cessation of routine vaccination, there is a rising population of unvaccinated people with little to no protection against* Orthopoxvirus* infections, including MPXV [1]. Additionally, there is a concern, due to the waning population immunity, that either VARV or MPXV could potentially be used as a bioterrorist weapon. Notably, MPXV caused an outbreak in the United States in 2003 due to importation of infected African rodents, which transmitted virus to pet black-tailed prairie dogs (*Cynomys ludovicianus*), causing human infection and demonstrating the ability of the disease to emerge outside of its normal ecological range [2, 3].

Based on clinical presentation, epidemiologic characteristics, geographic location, and genotyping, prior studies have identified 2 distinct MPXV clades, WA and CB [4, 5]. CB MPXV is associated with approximately 10% mortality and can be transmitted between humans; up to 6 sequential interhuman transmission events have been documented [6]. In comparison, WA MPXV is associated with milder disease, and person to person transmission has never been documented as the sole mode of transmission [7, 8].

Although our understanding of this important human pathogen continues to increase, the disease pathogenesis is still only partially described. MPXV causes human disease characterized by an approximately 13-day incubation period (average of 12–14 days) followed by a prodrome of fever for 1–3 days [9]. Following the prodrome, disseminated skin lesions begin to appear, developing first as macules then papules to vesicles to pustules. The pustules begin to scab approximately 4 weeks after initial infection. As seen in this study as well as previous prairie dog MPXV challenges, the prairie dog animal model also displays an incubation period of approximately 10–13 days followed approximately 2 days later by generalized cutaneous lesions [10, 11]. It has been challenging to truly identify a fever stage simply due to difficulties in temperature monitoring of the animals and their normal variations; however, a prodrome characterized by inappetence is consistently observed with this animal model. Skin lesions begin to scab approximately 24 days after onset. This model has allowed for the characterization of monkeypox disease including virus shedding (from the oral cavity shedding that begins prior to lesion onset), the occurrence of viremia, and finally the serologic detection of anti-*Orthopoxvirus* antibodies at or near the onset of lesion formation. Additionally, the prairie dog MPXV model has been utilized to describe observed differences between disease manifestations of the viral clades as well as comparisons of transmissibility of the 2 clades within this animal model [10, 12, 13]. We have also utilized the prairie dog MPXV model for the study of vaccine efficacy and antiviral benefit as well as for the study of potential pathogenic viral genes [14–16].

In the current study we challenged animals intranasally with an equivalent amount of Congo Basin (CB) or West African (WA) clade virus designed to cause symptomatic disease (8 × 10^3^ p.f.u.). Animals were then sacrificed at early time points after infection, as well as once symptomatic, in order to infer distribution of virus and characterize tissue pathology. Viral dissemination/loads and tissue pathology in 28 different tissues on days 2, 4, 6, 9, 12, 17, and 24 were analyzed and compared. Antigen distribution utilizing IHC and virologic analyses of these samples has confirmed differences in disease progression between the 2 clades of MPXV and identified sites that are important during early viral replication and cellular response to viral infection.

## 2. Materials and Methods

### 2.1. Animals

Wild-caught, juvenile black-tailed prairie dogs (PDs;* Cynomys ludovicianus*) were obtained from Colorado. At time of infection, animals were approximately 10 months old and had been prescreened by a veterinarian, determined to be in good health status, and found negative for the presence of anti-*Orthopoxvirus* antibodies. A sterile PIT tag was injected subcutaneously at the base of the neck for animal identification and noninvasive recording of body temperature. The average starting weight for animals challenged with WA MPXV was 781 grams (range 488–965), and the average for CB MPXV challenged animals was 740 grams (range 549–899). During experimental infections animals were housed individually in large (12.13′′ × 23.38′′ × 209.00′′) rat cages with aerosol filter tops. Cages were kept in a Duo-Flow biosafety cabinet in an animal Biological Safety Level-3 (ABSL-3) animal room. Animals were cared for in accordance with CDC Institutional Animal Care and Use Committee (IACUC) guidelines under an approved protocol (1683DAMPRAC). In addition to PD chow and hay, animals were provided with monkey biscuits for added dietary enrichment.

### 2.2. Viruses

The WA MPXV strain, MPXV-USA-2003-044, was isolated during the 2003 U.S. outbreak [3, 5] and the CB MPXV strain, MPXV-ROC-2003-358, was collected from a 2003 outbreak of MPXV in the Republic of Congo (ROC) [5]. Both viruses have been fully sequenced and underwent 2 passages in African green monkey kidney cells (BSC-40) prior to seed pool production. The seed pool preparations were then propagated in BSC-40 cells as previously described [2] and sucrose-cushion purified prior to being used for animal challenges.

### 2.3. Animal Inoculation

Inocula dosages (8 × 10^3^ plaque forming units (p.f.u.)) were calculated based on the morbidity and mortality rates observed in the author's previous study. Briefly, a challenge dose of 6 × 10^3^ p.f.u. WA MPXV or 8 × 10^3^ p.f.u. CB MPXV resulted in disease morbidity including skin lesions and viral shedding identified in oral cavity samples in 100% of animals challenged [11]. Both virus strain stocks were diluted in phosphate-buffered saline (PBS). Inocula titers were immediately reconfirmed by standard plaque assay (as described below). Animals were infected by an intranasal (IN) route of inoculation while under general anesthesia using 5% isoflurane administered through a VetEquip Incorporated vaporizer. For each virus strain, animals were inoculated with 1 of the virus clades in a total volume of 10 *μ*L (5 *μ*L in each nostril). Additionally, 7 animals were mock infected with PBS.

### 2.4. Observations and Sampling

Two animals challenged with virus from each MPXV clade as well as 1 PBS animal were preselected for euthanasia on day 2, 4, 6, 9, 12, 17, or 24 postinfection (p.i.). For all 24 days of study postinfection, individual animals were observed for signs of morbidity, fever, malaise (inappetence, decreased activity, recumbent with reluctance to move, etc.), and clinical lesions, including rash. On scheduled euthanasia days (see above), oropharyngeal swabs, weights, and lesion counts were collected from all animals while under general anesthesia (as described above), before subsets of animals were sacrificed by humane euthanasia. For those animals euthanized, blood and necropsy samples were also collected. Additionally, 1 animal from each MPXV clade was serially bled throughout the study for determination of antibody kinetics. Strict euthanasia criteria were applied throughout the study as follows: any animal that became unresponsive to touch, lost 25% or more starting body weight, or accrued a total score of 10 on the following scale was humanely euthanized; decreased activity (2 points); lethargy, unsteady gait, and inappetence (3 points each); labored breathing and recumbency (5 points each).

### 2.5. Necropsy and Tissue Specimen Collection

Necropsies on all animals were performed according to IACUC standards in an ABSL-3 laboratory and utilizing full ABSL-3 personal protective equipment (PPE). Samples taken during necropsy included eyelid, eye, inner cheek tissue, tongue, nasal cavity tissue, tonsils, submandibular lymph nodes/salivary glands, mesentery lymph nodes, oropharynx tissue, trachea, gallbladder, lungs, heart, spleen, pancreas, kidney, liver, duodenum, jejunum, ileum, stomach, brain, gonad, belly skin, lesion if present, feces, urine or bladder tissue, oral swab, ocular swab, and blood. Instruments were cleaned and decontaminated with 5% Microchem and 70% ethanol between collections of each tissue. Tissues were frozen at −70°C prior to further processing. Oral and ocular swabs were collected with sterile individual Dacron swabs and stored frozen without diluent. Serum was separated from whole blood and processed for serology and clinical chemistry levels (see below). Tissues and swabs were subsequently processed and further prepared for DNA analysis, virus isolation, histopathology, and immunohistochemistry (see below).

### 2.6. Sample Preparation for PCR and Viral Growth

Sample processing was performed under BSL-2 conditions with BSL-3 work practices. The BioRobot EZ-1 Workstation (Qiagen) was used for genomic DNA extraction of all blood, swab, and tissue samples. Samples were incubated with Qiagen buffer and proteinase K at 55°C for an hour to degrade tissue and inactivate viable virus particles prior to DNA extraction. For whole blood samples, 100 *μ*L was used for DNA extraction, and the remaining blood was used for tissue culture propagation. For each swab collected, 400 *μ*L of PBS was added. The swab extraction tube systems (SETS) (Roche) protocol was used to recover sample from the swab. DNA was extracted from 100 *μ*L of the swab lysate. The remaining swab eluate was used for virus isolation. For tissue preparation, 1 mL aliquots of PBS and SPEX bead (SPEX Sample Prep) were prepared. The PBS/bead aliquot was then poured into a tube containing the individual tissue sample. The GenoGrinder 2000 (SPEX Sample Prep) was used following the manufacturer's instructions to create a tissue homogenate. 100 *μ*L of the homogenate was extracted for DNA isolation. The remaining homogenate was used for virus isolation.

### 2.7. Real-Time PCR Analysis

Samples were tested by real-time PCR using forward and reverse primers and probes complimentary to the conserved non-VARV* Orthopoxvirus* (OPXV) E9L (DNA polymerase) gene [17]. Purified MPXV DNA (10 fg–1 ng) was used as standard controls to allow quantification of viral DNA. A sample was considered positive if duplicate reactions showed amplification crossing the threshold at cycle (CT) of 37 or earlier. A weakly positive sample displayed CT values 38-39 (duplicates).

### 2.8. Virus-Tissue Infectivity

All samples were stored at −70°C until virus isolation was attempted. Previous analyses demonstrated that real-time PCR detection is more sensitive than viability assays due to the ability to detect trace amounts of MPXV DNA in samples without viable virus [2]. Therefore, specimens were first tested for presence of OPXV DNA by PCR and, if positive, were subsequently evaluated for viable virus by tissue culture propagation. Each swab or tissue sample was titrated in duplicate using 10-fold dilutions of swab eluate or tissue slurry on BSC-40 cell monolayers, incubated at 35.5°C and 6% CO_2_ for 72 hours and subsequently stained with crystal violet and formalin to visualize plaques. Titers were expressed as p.f.u. per milliliter of blood or swab eluate, or p.f.u. per gram of tissues.

### 2.9. Serologic Analysis

A modified ELISA was used for analysis of anti-OPXV immunoglobulin types M and G in separated serum as previously described in detail [10, 18].

### 2.10. High Content Screening-Green Fluorescent Protein (HCS-GFP) Neutralization Assay

All serum samples that were considered negative by ELISA were further tested with the HCS-GFP neutralization assay as described in Keckler et al. In brief, serum samples were serially diluted from 1 : 40 to 1 : 1280 and then neutralizing antibody (NAb) titers against vaccinia virus were measured using a GFP based assay. The HCS-GFP assay detects the percentage of GFP-producing responder cells (R), and this value is then normalized to control wells to produce the relative percent responders (RPR) titer. The reported values in this paper are 50% RPR titers that are equivalent to the serum dilution that neutralizes 50% of viral infection (ID_50_) in a traditional plaque reduction neutralization titer (PRNT) assay. The 50% RPR titer was calculated using a modified variable slope sigmoidal equation (Hill equation, Levenberg Marquardt algorithm) and Prism 5.0 software (GraphPad) with goodness of fit to this sigmoidal curve (represented by *R* ^2^; all *R* ^2^ values considered positive were above 0.9000) calculated by the least-squares method.

### 2.11. Blood Chemistry Analysis

Serum was separated from whole blood, transferred to a clean tube, and stored at −20°C prior to analysis. The Piccolo blood chemistry analyzer (Abaxis) was utilized to determine the following blood chemistry profiles: sodium (NA), potassium (K), total carbon dioxide (Tco2), chloride (CL), glucose (GLU), calcium (CA), blood urea nitrogen (BUN), creatinine (CRE), alkaline phosphatase (ALP), alanine aminotransferase (ALT), aspartate aminotransferase (AST), total bilirubin (TBIL), albumin (ALB), and total protein (TP).

### 2.12. Histopathologic, Immunohistochemical, and Electron Microscopy Studies

After euthanasia, a necropsy was performed and tissues were taken as described above. Pieces of each tissue were fixed in 10% neutral-buffered formalin for at least 48 hours and then transferred to 70% ethanol for routine processing to create paraffin block which were subsequently sectioned at 3 *μ*m. Routine hematoxylin-eosin (H&E) stains were performed for histopathological evaluation. Immunohistochemical tests using a multistep immunoalkaline phosphatase technique were performed on sections using a previously described technique [19]. The primary antibody used for this test was an in-house generated rabbit polyclonal anti-monkeypox or anti-VARV hyperimmune sera antibody. For ultrastructural analysis, hematoxylin and eosin stained sections were processed for thin-section electron microscopy (EM). Briefly, sections were prepared on-slide and processed through a graded ethanol series to rehydrate the tissue for osmium tetroxide fixation. Tissue was then block stained with uranyl acetate and rinsed with water. The sample was microwave processed with ethanol to dehydrate, followed by acetone to prepare the tissue for resin infiltration. Following 4 exchanges of resin, the tissue was polymerized in a final exchange of resin at 60°C. Thin sections were cut and stained with uranyl acetate and lead citrate before viewing sections at the electron microscope (Tecnai Spirit, FEI, Hillsboro, OR).

### 2.13. Specialized IHC Assays

#### 2.13.1. Apoptosis Assay

For detection of apoptosis ApopTag Peroxidase In Situ Apoptosis Detection Kit (Millipore) was used according to the manufacturers' instructions. This kit consists of terminal deoxynucleotidyl transferase mediated deoxyuridine triphosphate nick-end labeling (TUNEL) methodology. Modifications of the manufacturers' protocol included the use of Vector NovaRed horseradish peroxidase (HRP) substrate (Vector Labs) in place of DAB as the chromogen. Counterstaining was performed with Gill's hematoxylin. Counting apoptotic cells was performed by randomly moving each slide until 10 adjacent fields were counted. These 10 fields of view were counted by 2 individuals.

#### 2.13.2. Double Stain

For visualization of apoptosis and viral antigen colocalization, a sequential IHC protocol was followed. First, the apoptosis assay (HRP) was performed as described above. Modifications for this described assay included an additional blocking step with Dual Endogenous Block (Dako) following the hydrogen peroxidase incubation. Following incubation with the Vector NovaRed chromogen, slides were washed in ddH20. The slides were then incubated with an in-house generated rabbit polyclonal anti-VARV hyperimmune sera antibody (1 : 5000). The secondary antibody that was then utilized was a swine anti-rabbit alkaline phosphatase purified antibody (Dako). BCIP/NBT (Dako) was then used as the chromogen to create a blue-black color in the presence of viral antigen. Counterstaining for this double-stain IHC assay was done with a nuclei methyl green stain. Slides from PBS inoculated prairie dogs were used as negative controls.

### 2.14. Data Analyses

As data were not normally distributed, nonparametric statistical analyses were used [20]. The Wilcoxon rank-sum test was utilized to compare the blood chemistry percent change (Percent change from day zero value to necropsy day value) for the WA MPXV, CB MPXV, and PBS control animals, controlling for necropsy day. The p.f.u. value for each tissue type was compared between the CB MPXV and WA MPXV infected prairie dogs, controlling for day of necropsy using the Wilcoxon rank-sum test. A *p* value of ≤0.05 was considered statistically significant. Data analysis was performed using SAS v9.3.

## 3. Results

### 3.1. WA MPXV Clade: Clinical Observations and Gross Necropsy Findings

Animals appeared healthy, without objective signs or symptoms of illness until day 9. Gross observations of tissues from animals sacrificed on days 2, 4, and 6 were similarly indistinguishable from the control animals (Table 1). Beginning on day 9, the majority of infected animals displayed respiratory depression while under anesthesia. Other clinical symptoms and signs observed included inappetence, maculopapular cutaneous lesions forming on the medial aspect of the pelvic limbs of 2 animals, and nasal discharge in 1 animal. During necropsy of PD15 on day 9, the kidneys were pale; the other animal euthanized at this time (PD16) was grossly normal. All remaining animals challenged with WA MPXV were observed to have disseminated cutaneous papules and/or vesicles by day 12 p.i (Table 1). Unexpectedly on day 12 p.i., PD21 expired from infection; this animal was noted to have nasal discharge and inappetence prior to death. During necropsy of PD21, hepatomegaly was noted as was bilateral hemorrhage on the horns of the uterus and the adipose tissue associated with the round ligament. Additionally, there were hyperemic foci associated with the ovaries and oviducts. On day 12, PDs 17 and 18 presented with submandibular lymphadenopathy and additionally, for PD17, hyperemic foci associated with the oviducts. PD 19 was necropsied on day 17 p.i.; inappetence had been noted during infection but the activity of this animal appeared normal. Gross observations included lymphadenopathy, hemorrhagic foci in the adipose tissue associated with lymph nodes, and multiple, small foci of hemorrhage on the visceral pleura of the lungs. On day 24, all cutaneous lesions had resolved at the time of necropsy for PD22 and PD20; however, pericarditis was observed in PD22. With the exception of disseminated skin lesions, morbidity for these 2 animals during the course of the study was not observed.

### 3.2. CB MPXV Clade: Clinical Observations and Gross Necropsy Findings

Clinically, all animals were healthy without discernible signs and symptoms of illness until day 9 (Table 2); in contrast, animals euthanized on scheduled day 4 manifest with foci of serosal congestion in the stomach and colon (PD25), or foci of pleural congestion and serosal congestion in the stomach (PD26). On day 6 p.i. all animals were asymptomatic and grossly normal. As seen with the WA MPXV challenged animals, beginning on day 9 the majority of infected animals displayed respiratory depression while under anesthesia. Also observed on this sample day, 3 animals developed disseminated maculopapular cutaneous lesions and inappetence was noted for 1 animal. PD29 was necropsied on day 9 and was asymptomatic with no gross abnormalities. PD30 had developed cutaneous lesions (maculopapular or papular) and had nasal discharge; this animal died while under anesthesia. During necropsy, the lungs had multiple, randomly distributed foci of parenchymal hemorrhage. By day 12 p.i., 5/6 of the remaining animals had developed papular and/or vesicular cutaneous lesions; lesions were observed on the sixth animal by day 16 p.i. PD32 expired due to infection on day 12. This animal had developed inappetence, facial edema, and cutaneous lesions before death. Necropsy of this animal revealed multiple hemorrhagic foci in the jejunum and submandibular lymphadenopathy. The other animal necropsied on this day (PD31) also had facial edema and disseminated papular cutaneous lesions; necropsy results of internal organs were grossly normal. On day 13 p.i., PD36 had to be euthanized before the preselected euthanasia date due to measures of morbidity. This animal had nasal discharge and inappetence beginning on day 10 p.i. By day 12, the animal presented with papular/vesicular cutaneous lesions, periocular erythema and epiphora associated with 1 eye, purulent discharge from the nares, and a distended abdomen. During necropsy, gastrointestinal tract gas distention was observed. The uterus had foci of serosal hemorrhage; multiple hemorrhagic foci were observed in the periovarian adipose tissue. Mucosal associated lymphoid tissue (MALT) was hemorrhagic. Additionally, the cardia of the stomach showed mucosal hemorrhage. Both animals necropsied on day 17 (PDs 33 and 34) had facial edema that was diminishing at time of necropsy and purulent nasal discharge as well as crusted cutaneous lesions. Necropsy of PD33 revealed a focus of hemorrhage in the jejunum and multiple hemorrhagic foci were observed in the periovarian adipose tissue. PD34 appeared grossly normal. On the final time point, day 24 p.i., PD35 was recovering from infection. All cutaneous lesions were healing at time of necropsy and no abnormalities involving internal organs were seen during necropsy of this animal.

### 3.3. PBS Control Animals: Clinical Observations and Gross Necropsy Findings

With regard to the 7 animals that were mock infected with PBS, 1 control animal was euthanized at each time point. None of these animals showed clinical symptoms indicative of MPXV infection during the course of the study. Additionally, gross necropsy observations did not indicate viral infection and all 7 animals were negative for viral DNA and OPXV antibodies.

### 3.4. Blood Chemistry Results

The mean percent value change from day zero to necropsy for each blood chemistry was compared between WA MPXV infected animals, CB MPXV infected animals, and PBS controls, controlling for day of necropsy (Supplementary Table  1 in Supplementary Material available online at http://dx.doi.org/10.1155/2015/965710). No significant changes were observed in these blood chemistry values (*p* values range 0.1–1). Sera from the 2 animals that died due to MPXV infection during the study were not available for analysis (PDs 21 and 32). However, when comparing pre- and postblood chemistry values in the animal euthanized on day 13 p.i. (PD36) liver enzyme levels were abnormal (increased alanine aminotransferase and decreased albumin and alkaline phosphatase), as we have seen in previous studies [12, 13]. Additionally glucose levels were elevated in PD36 most likely due to stress and/or the moribund state of the animal and blood urea nitrogen and sodium levels were increased.

### 3.5. Antibody Response

**Figure 1:**
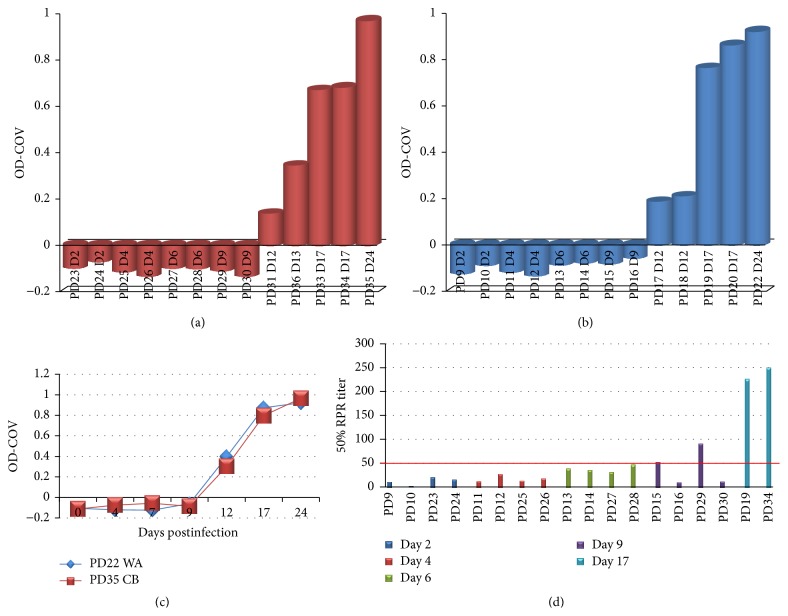
Antibody response in MPXV challenged prairie dogs. Groups of prairie dogs were serially sacrificed (days 2, 4, 6, 9, 12, 17, and 24) following intranasal challenge (8 × 10^3^ pfu) with Congo Basin (CB) or West African (WA) MPXV. Antibody response at time of sacrifice by ELISA is shown for individual MPXV infected prairie dogs (CB (a) and WA (b)). Kinetics of antibody titers for two animals that were serially bled throughout the study are shown in (c). Neutralizing titers for animals sacrificed on or before day 9 as determined by HCS-GFP are shown in (d); two animals sacrificed on day 17 p.i. serve as positive controls.

All serum samples collected from prairie dogs were negative by ELISA until day 12/13 p.i., at which time all 4 sacrificed animals had detectable orthopoxvirus antibodies (PDs 21 and 32 did not have serum available for analysis) (Figures 1(a) and 1(b)). Two animals that were serially bled throughout the study are used to illustrate the dramatic increase in antibody titer between days 12 and 17; levels appear to be beginning to plateau on day 24 p.i. Figure (Figure 1(c)). Although no antibodies were detected by ELISA before day 12, 2 animals sacrificed on day 9 (PD15 (WA MPXV) and PD29 (CB MPXV)) were shown to have neutralizing antibodies (Figure 1(d)). Antibody titers and neutralizing antibody levels were similar when comparing animals challenged with the 2 MPXV clades.

### 3.6. Kinetics of Viral Spread

#### 3.6.1. Viremia

**Figure 2:**
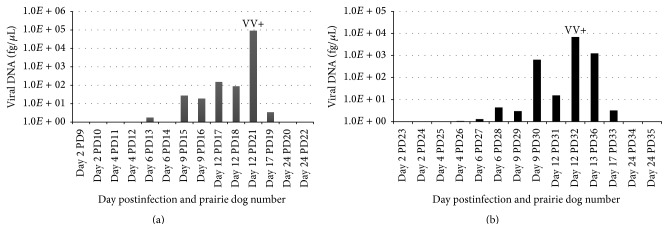
MPXV challenged prairie dog viremia. Groups of prairie dogs were serially sacrificed (days 2, 4, 6, 9, 12, 17, and 24) following intranasal challenge (8 × 10^3^ pfu) with West African (a) or Congo Basin (b) MPXV. Viremia was determined by viral DNA in blood samples. Only blood from prairie dogs 21 and 32 was positive for viable virus (VV) (1.8 × 10^6^ pfu/mL and 2 × 10^6^ pfu/mL, resp.). Values are shown as DNA (fg/*μ*L) on a log scale.

Viral DNA in blood samples was detectable from 3/4 animals on day 6 (2 CB MPXV and 1 WA MPXV infected animals) and all 4 euthanized animals on day 9 p.i. (Figure 2). The highest loads of viral DNA in the blood were seen on day 12; by day 24 p.i. viral DNA was undetectable in all blood samples tested. Viable virus was only detected from 2 blood samples collected on day 12 from the 2 animals that perished due to infection (PDs 21 (WA MPXV) and 32 (CB MPXV); Figure 2). Both of these animals had values of approximately 2 × 10^6^ p.f.u./mL of blood, suggesting that when viral loads are below this amount, we are unable to detect viable virus. A previous study has indicated that the methods utilized may not be sensitive enough to detect low levels of intact virions within blood samples [13].

#### 3.6.2. WA MPXV Clade: Kinetics of Viral Spread

**Figure 3:**
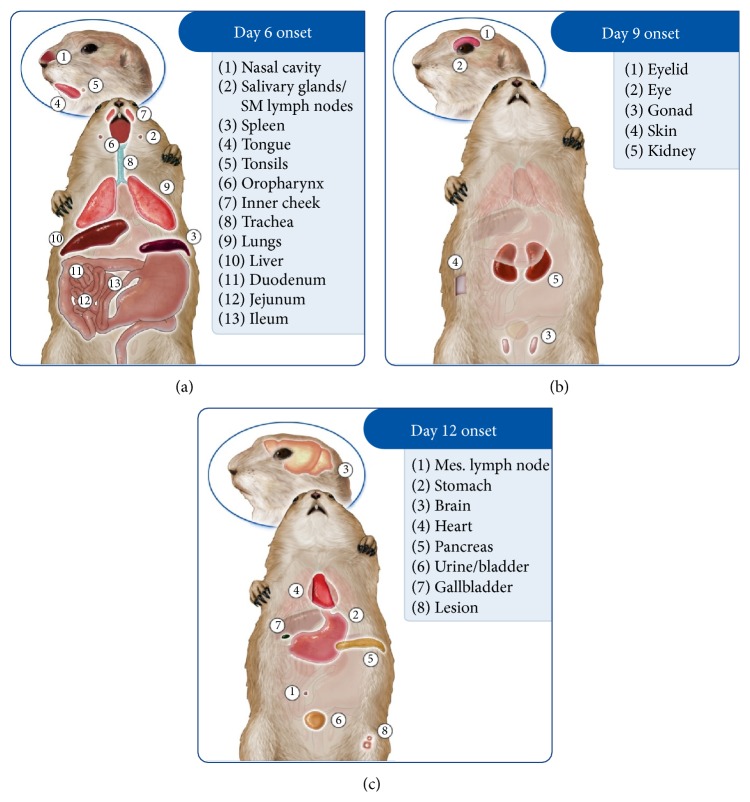
Schematic depicting WA MPXV disease progression in a prairie dog. Groups of prairie dogs were intranasally challenged (8 × 10^3^ p.f.u.) with West African (WA) MPXV and serially sacrificed (days 2, 4, 6, 9, 12, 17, and 24). Samples were evaluated for the presence of virus and were grouped by initial detection of viable virus (day 6 (a), 9 (b), or 12 (c) p.i.).

**Figure 4:**
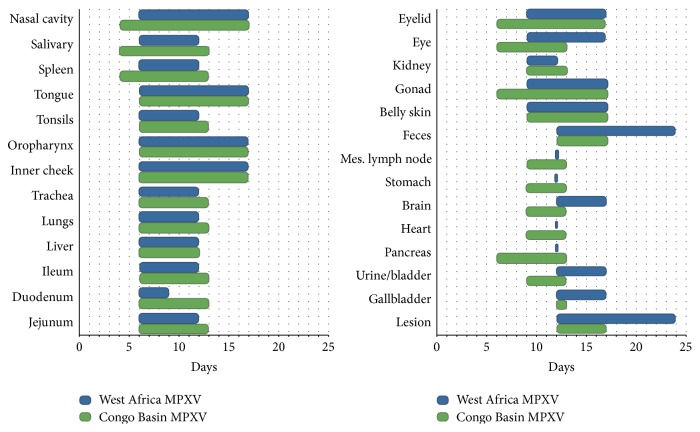
Monkeypox viral trafficking within prairie dogs. Groups of prairie dogs were serially sacrificed (days 2, 4, 6, 9, 12, 17, and 24) following intranasal challenge (8 × 10^3^ p.f.u.) with West African (blue) or Congo Basin (green) MPXV. Samples were evaluated for the presence of virus by tissue culture. The time courses of viral shedding from each tissue type are shown above.

On day 2 p.i. viral DNA was only detected in the submandibular lymph node/salivary gland from those animals challenged with WA MPXV. By day 4 additional tissues were positive for viral DNA including the tongue, nasal cavity, tonsils, oropharynx, lungs, heart, spleen, liver ileum, and stomach in at least 1 animal necropsied (Table 3). However, viable virus was not detected in any tissues until day 6 from those animals challenged with WA MPXV (Figures 3 and 4 and Table 3). Those tissues positive beginning on day 6 included nasal cavity, submandibular lymph nodes, spleen, and tonsils as well as additional tissues as summarized (Figure 3). Of the sacrifice days chosen for this study, all tissues tested from the WA MPXV challenged animals were classified by onset of detectable viable virus: day 6, day 9, or day 12. The majority of tissues were positive for virus beginning on day 6 (Figures 3 and 4). Viral loads tended to be highest from tissues taken on day 12 p.i.; however nasal cavity yielded the highest level of virus (7.7 × 10^8^) compared to all other tissues on day 9, followed closely by liver (5.7 × 10^8^) and spleen (4.9 × 10^8^) tissues on day 12 (Table 3, Supplementary Material Figure S1).  Figure 4 illustrates the tissues that were positive for virus on each sample day for both MPXV clades. Noticeably, the mesenteric lymph nodes, stomach, heart, and pancreas were only positive on day 12 p.i. for WA MPXV challenged animals, unlike the CB MPXV challenged animals. Urine/bladder tissue and feces were also analyzed for virus, and the WA MPXV challenged animals seem to shed virus longer from these samples compared to CB MXPV (Figure 4). On the last sample day (day 24 p.i.), 80% of the tissues were still positive for viral DNA, but only the lesion and fecal samples yielded viable virus (Table 3, Figure 4).

#### 3.6.3. CB MPXV Clade: Kinetics of Viral Spread

**Figure 5:**
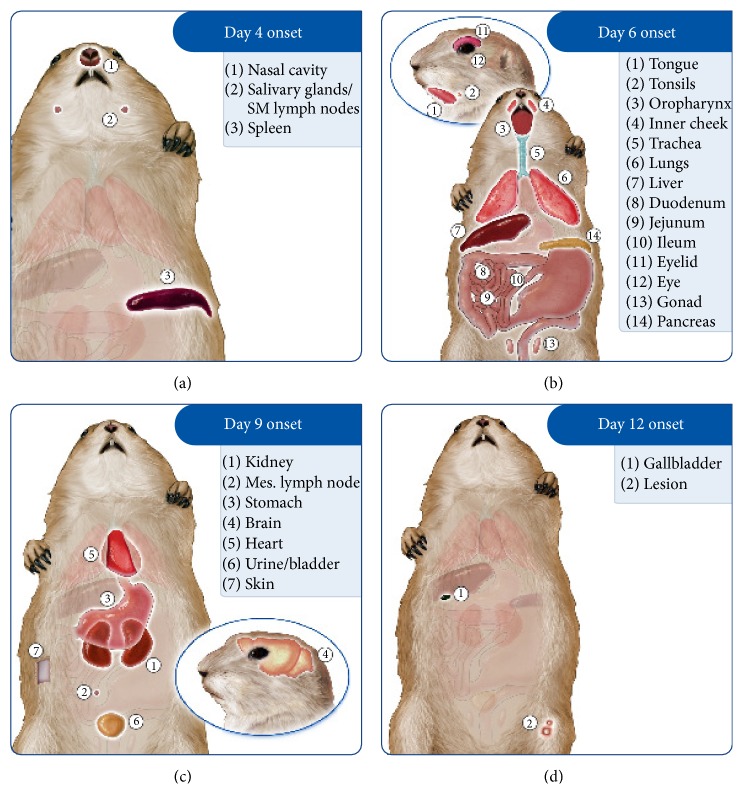
Schematic depicting CB MPXV disease progression in a prairie dog. Groups of prairie dogs were intranasally challenged (8 × 10^3^ p.f.u.) with Congo Basin (CB) MPXV. Groups of prairie dogs were serially sacrificed (days 2, 4, 6, 9, 12, 17, and 24) following challenge. Samples were evaluated for the presence of virus and were grouped by initial detection of viable virus (day 4 (a), 6 (b), 9 (c), or 12 (d) p.i.).

Beginning on day 2 p.i., low levels of viral DNA were detected from 1 animal in eyelid, nasal cavity, oropharynx, and liver samples (Table 3). By day 4 p.i., 57.9% of tissues tested were positive for viral DNA, and unlike WA MPXV challenged animals in which no samples yielded viable virus on day 4 p.i., the nasal cavity, submandibular lymph node/salivary gland tissue, and spleen samples yielded viable virus from CB MPXV challenged animals (Figures 4 and 5, Table 3). Therefore, for the CB MXPV challenged animals, samples were classified into day 4, day 6, day 9, or day 12 viable virus onsets. As was seen for WA MXPV challenged animals, the majority of tissues were positive beginning on day 6. The majority of tissues had the highest viral load on day 12 similar to WA MXPV infected animals; for CB MPXV infected animals the liver (2.9 × 10^9^) yielded the highest load followed by spleen, nasal cavity, and lesion all on day 12 p.i., respectively (7.5 × 10^8^, 2.6 × 10^8^, and 2.3 × 10^8^) (Table 3, Supplementary Material Figure S2). At study end (day 24 p.i.), less than half of the tissues had detectable levels of viral DNA, and no samples were positive for viable virus from those animals challenged with CB MPXV.

### 3.7. Pathology Findings

#### 3.7.1. Histopathologic, Immunohistochemical, and Electron Microscopy Findings

MPXV infected prairie dogs exhibited histologic changes attributable to viral infection beginning on day 6. Early changes for both MPXV clades were seen within the spleen which had prominent neutrophils within red pulp and increased apoptotic or necrotic cells in all 4 euthanized animals. Apoptotic or necrotic cells were seen in the lung from 1CB MPXV infected animal; 1 WA MPXV infected animal had prominent bronchus associated lymphoid tissue within the lung on day 6 p.i. Beginning on day 9 p.i., histopathologic changes attributable to viral infection were observed in 1 or more animal at each time point and those data were compiled in the text below for each day and clade of virus. Additionally, IHC positive results were not obtained until day 9 p.i. from any infected animals and are summarized in Tables 1 and 2.

#### 3.7.2. WA MPXV Clade Pathology Findings

On day 9 p.i. dermal lesions characterized by epidermal spongiosis and vacuolation predominantly in the basal layer were observed. Superficial and mid dermal inflammation was composed predominantly of neutrophils and surrounding vessels had reactive endothelium and walls with edema, fibrin, and acute inflammatory infiltrates. Small dermal vessels were occasionally obscured by necrotic debris admixed with fibrin. Lymph nodes had superficial cortical and subcapsular necrosis that variably extended into adjacent soft tissues, which were edematous with small numbers of neutrophils and immature or reactive fibrosis. Necropsy results for animals on day 12 p.i. revealed cutaneous lesions characterized by small foci of epidermal vacuolation with minimal exocytosis of neutrophils. Oral and pharyngeal mucosal epithelia had small foci of acute necrosis. The soft tissues adjacent to the uterus of 2 animals showed necrosis within or adjacent to early fibrosis. One animal also had accompanying myometrial necrosis. The liver had multiple foci of randomly distributed, occasionally coalescing foci of necrosis accompanied by neutrophilic inflammation. Hepatocytes frequently had intracytoplasmic, amphophilic inclusion bodies that were round and ranged from 3 to 6 micrometers in diameter. Regional necrotizing enteritis, multiple foci of splenitis, and a focal keratitis were observed. On day 17 p.i., animals infected with WA MPXV had cutaneous lesions characterized by multiple foci in the dermis composed of necrotic cellular debris. Peritesticular soft tissues had foci of necrosis with minimal fibrosis and chronic-active inflammation. At the last time point, day 24 p.i., the skin of 1 animal showed focal dermal fibrosis covered by a hyperkeratotic epidermis.

#### 3.7.3. CB MPXV Clade Pathology Findings

Animals euthanized on day 9 p.i. had cutaneous lesions characterized by small foci of epidermal cell vacuolation. Multiple dermal foci showed acute inflammation that was predominantly perivascular. The small intestinal lymphoid tissue (MALT) showed focal necrosis. The spleen had multiple, discrete necrotic foci. Interstitial edema with minimal hemorrhage was observed in the lungs. On days 12 and 13, animals had cutaneous lesions characterized by foci of epidermal necrosis with serocellular crusts. The dermis showed acute, predominantly perivascular inflammation. The rostral nasal cavity had epithelial necrosis and ulceration with acute inflammatory infiltrates and abundant luminal necrotic debris. The oral and pharyngeal mucosa had multiple foci of epithelial necrosis and ulceration with acute inflammation in the adjacent submucosa. The liver had multiple foci of randomly distributed, occasionally coalescing foci of necrosis accompanied by neutrophilic inflammation. Hepatocytes frequently had intracytoplasmic, amphophilic inclusion bodies that were round and ranged from 3 to 6 micrometers in diameter. Lymph nodes were diffusely necrotic and surrounding soft tissues had early fibrosis. On day 17, resolving foci of superficial dermatitis and foci of epidermal necrosis was observed. Chronic sinusitis was observed in the rostral nasal cavity. Similar to WA MPXV infected animals, on day 24 p.i., cutaneous lesions were the predominant finding and characterized by dermal fibrosis, hyperkeratosis, and crusting.

**Figure 6:**
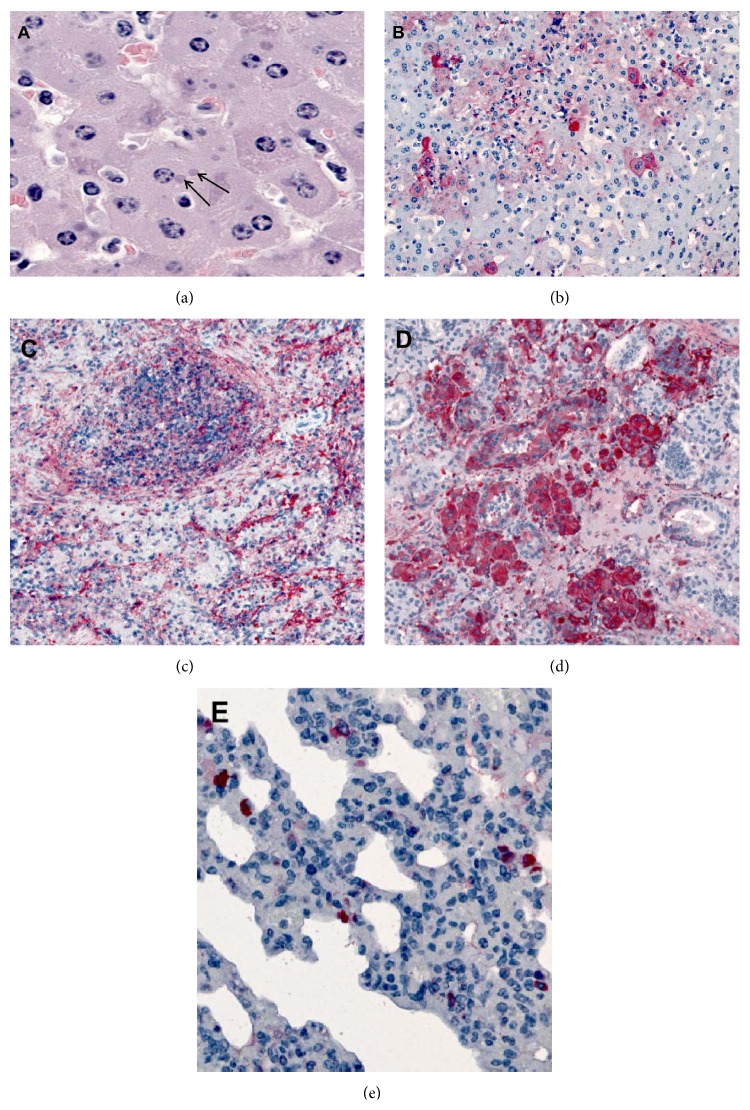
Histopathologic and immunohistochemical findings in a prairie dog that succumbed to West African MPXV (PD21). (a) Liver showing typical intracytoplasmic basophilic inclusions (arrows) by H&E staining and (b) multifocal staining of* Orthopoxvirus* antigens in hepatocytes by IHC. (c–e) Extensive staining of* Orthopoxvirus* antigens in spleen (c), salivary gland (d), and lung (e).

**Figure 7:**
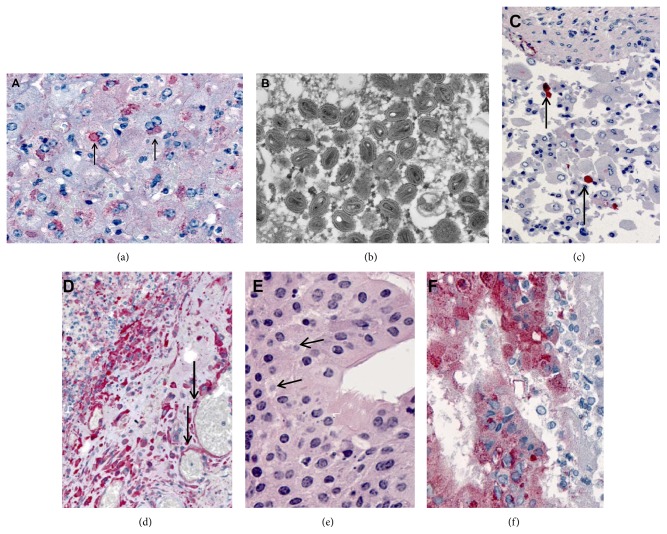
Histopathologic, ultrastructural (EM), and immunohistochemical findings in a prairie dog that succumbed to Congo Basin MPXV (PD32). (a) Liver showing prominent inclusions (arrows) as seen by IHC staining of* Orthopoxvirus* antigens. (b) Thin section EM of liver showing oval and brick shaped monkeypox virions, with dense dumb-bell shaped cores within hepatocytes. (c) Urinary bladder showing desquamated epithelial lining cells (arrows) containing poxvirus antigens as seen by IHC. (d) Submandibular lymph node showing abundant macrophages and vascular (arrows) poxvirus antigens seen by IHC. (e-f)* Orthopoxvirus* IHC showing typical intracytoplasmic inclusions (arrows) by H&E (e) and extensive staining of orthopoxvirus antigens as seen by IHC (f) within the oropharynx.

Comparing pathology of 2 animals that succumbed to disease on day 12 from each MPXV clade, both animals demonstrated abundant viral antigen (indicated by red chromogen) in all organs except the brain. Liver sections from PD21 that died from WA MPXV had typical intracytoplasmic basophilic inclusions and multifocal staining of orthopoxvirus antigens in hepatocytes (Figures 6(a) and 6(b)). Extensive staining of orthopoxvirus antigens was seen in the spleen, salivary gland, and lung (Figures 6(c) and 6(d)). Additionally, mesenchymal cells in the myometrium and broad ligament were positive for antigen as was the small intestinal staining in lamina propria fibroblasts and epithelial cells (Table 1). PD32 which died after CB MPXV challenge similarly had prominent inclusions within the liver (Figure 7(a)). Thin section EM of liver from this animal showed oval and brick shaped MPX virions, with dense dumb-bell shaped cores within hepatocytes (Figure 7(b)). The urinary bladder showed desquamated epithelial lining cells (arrows) containing poxvirus antigens (Figure 7(c)) and the submandibular lymph node had abundant macrophages and vascular (arrows) poxvirus antigens seen by IHC (Figure 7(d)). Orthopoxvirus IHC showed typical intracytoplasmic inclusions (arrows) by H&E (Figure 7(e)) and extensive staining of orthopoxvirus antigens within the oropharynx (Figure 7(f)).

#### 3.7.4. Virus Induced Apoptosis

**Figure 8:**
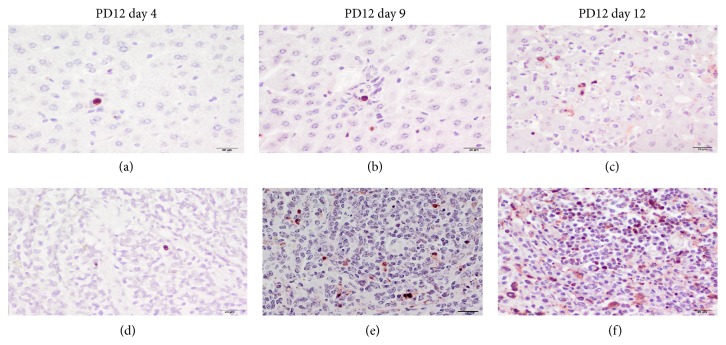
Apoptosis within MPXV infected prairie dog liver and spleen. Prairie dogs intranasally challenged (8 × 10^3^ p.f.u.) with West African WA MPXV were serially sacrificed (days 2, 4, 6, 9, 12, 17, and 24) following challenge. Representative images of immunohistochemical staining of paraffin-embedded prairie dog liver (a, b, c) and spleen (d, e, f) sections stained with Millipore Apoptosis kit. Apoptotic cells are identified by red-brown staining nucleus. (400x original magnification, scale bar = 20 *μ*m, immunoperoxidase method, Vector NovaRed chromogen, hematoxylin counterstain).

Individual liver and spleen sections were screened for apoptosis utilizing a TUNEL assay. These tissues that were chosen as the spleen seem to be an early target of infection, while the liver is an intermediate/late target. Additionally, as described above for both MPXV clades, the liver and spleen have appreciably high loads of virus during the course of infection (Table 3, Supplementary Material Figures  1 and  2). For both WA and CB challenged animals the number of apoptotic cells within the liver only increased slightly and was comparable between the clades (Figure 8, Supplementary Material Table S2). Kupffer cells were only observed to stain for apoptosis in the liver of 1 animal; instead hepatocytes were the predominant cell type that was positive for apoptosis. Unlike the liver, there was a noticeable trend of increased apoptosis in spleen sections (Figure 8) which was especially appreciable within the CB MPXV challenged animals (Supplementary Material Table  2). When comparing the 2 animals that were found dead from MPXV infection on day 12 (WA MPXV PD 21 and CB MPXV PD 32), PD32 had greater than 6 times the number of apoptotic splenocytes compared to PD21.

**Figure 9:**
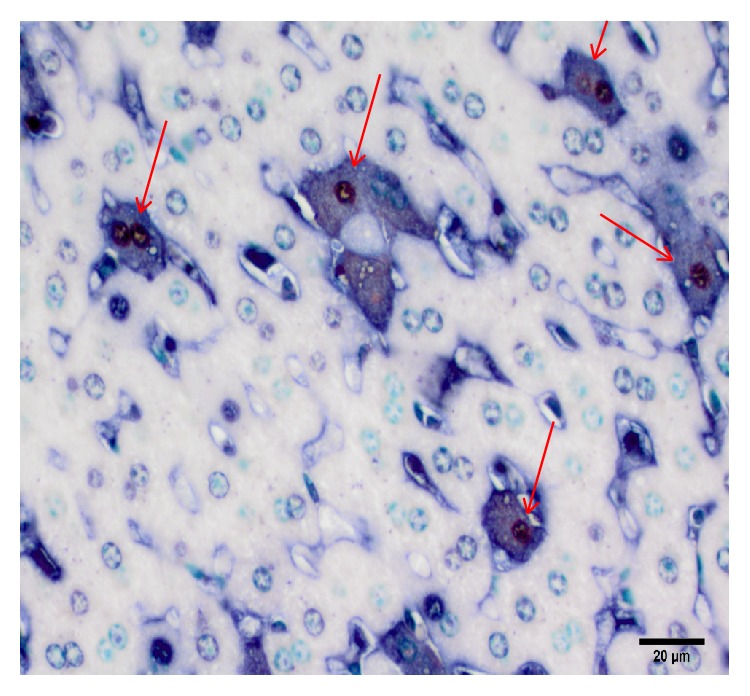
Monkeypox virus colocalizes with apoptotic cells. Representative liver section from Congo Basin MPXV infected prairie dog on day 12 p.i. Apoptotic cells (red-brown nuclei) tended to also stain for poxvirus (blue-black cytoplasmic staining, arrows). (Immunoperoxidase method with Vector Red chromogen, alkaline phosphatase method with BCIP/NBT Chromogen; 400x original magnification, scale bar = 20 *μ*m; methyl green counterstain.)

A double IHC assay was performed on a small subset of known MPXV positive liver and spleen sections to investigate whether the virus colocalizes with the apoptotic cells. As demonstrated in Figure 9, we did observe colocalization of the virus (blue-black stain) in the majority of cells that were also positive for apoptosis (red-brown nuclei) in both liver and spleen sections.

#### 3.7.5. Coinfection with Giardia or Coccidia

Although animals were inspected by a licensed veterinarian, as well as treated for fleas and intestinal worms upon capture and arrival at CDC facilities, it was noted during histological examination that a portion of the animals were infected with either of 2 protozoan parasites, giardia (*n* = 9/33) or coccidia (*n* = 3/34). Both of these organisms are common parasites found in many wild rodents [21–25], and likely this is also the case for wild prairie dogs. Our data does not suggest that infection with either giardia or coccidia had a negative impact on the health of the animals or the disease progression of MPXV. The range in starting weights of the WA MPXV challenged animals was 488–965 grams; CB MPXV starting range was 549–899 grams. In comparison, the 9 animals that were positive for giardia had a starting weight range of 538–965 grams. The weight range for the 3 animals that were positive for coccidia was 748–802 grams. Thus, the animals with parasites did not have appreciably low weights compared to parasite-free animals. The 2 animals that unexpectedly perished from MPXV infection as well as the prairie dog that had to be euthanized due to morbidity criteria (PDs 21, 32, and 36) were negative for both giardia and coccidia. Additionally, there were no observed differences in the development of antibody titers between animals coinfected with parasites and those that were parasite-free. Furthermore, no noticeable symptoms were observed in the animals that were found to be positive for coccidia or giardia such as diarrhea during the course of the study.

## 4. Discussion

Similar onset and range of clinical symptoms were observed in animals challenged with virus representatives of the WA and CB MPXV clades. Although we used the same inoculum for each MPVX clade (8 × 10^3^ p.f.u.), it is worth noting that we previously determined that CB MPXV has approximately a hundred times lower LD50 value than the WA MPXV clade (5.9 × 10^3^ versus 1.29 × 10^5^, resp.) with this animal model [11]. The 2 unscheduled deaths in the CB MPXV group compared to the 1 unscheduled WA MPXV death is explained by this difference. However, because the current study was designed to look at kinetics of viral spread* in vivo* using a serial sacrifice technique, the study was not designed to determine differences in mortality between the clades.

By day 9 p.i., respiratory depression while under anesthesia, as well as inappetence, formation of cutaneous poxvirus-lesions, and nasal discharge, occurred within multiple animals. These clinical signs, including respiratory depression under anesthesia were only seen with infected animals and not with the PBS controls. Inhalation anesthetics are eliminated from an animal primarily from the lungs, liver, and kidneys and injury to these organs, such as through viral infection, may result in dose-related complications, including respiratory depression. As by day 9 p.i., the kidney, liver, and lung tissues of infected animals had high loads of virus, it is possible that the viral induced organ damage resulted in decreased clearance of the anesthetic and respiratory depression of the animal and resulting respiratory depression. During peak infection (day 12 p.i.), however, CB MPXV challenged animals developed more pronounced signs of morbidity including facial edema, gastrointestinal distention, and ocular erythema and epiphora. Blood chemistry results did not display significant changes throughout the study; however the animal that was euthanized had abnormalities suggesting liver injury and dehydration. Based on the extremely high loads of virus recovered from the liver tissue, it is reasonable to assume that there was viral-induced tissue injury. The earlier viral kinetics of CB MPXV as well as the increased tissue burden of virus in these infected animals compared to WA MPXV infected animals could account for the increased morbidity and mortality associated with the CB clade of MPXV. When comparing peak mean viral loads in the tissues harvested, 74% of tissues had higher peak loads from the CB infected animals compared to WA infected animals (Table 3). However *p* values for these comparisons were not statistically significant when comparing each tissue type (CB versus WA) and controlling for day of necropsy (*p* value range of 0.21–1; data not shown). Of note, in animals infected with virus from either clade, day 12 p.i. seems to be a pivotal time in this animal model. On day 12/13 p.i. unexpected deaths were observed, antibody production was uniformly observed, peak virus levels are seen, and the highest percent of viral isolates from tissues are obtained. Furthermore this was also the only point at which viable virus was recovered from blood samples.

Viremia as detected in the form of viral DNA was first detected on day 6; this was after the detection of viral DNA/infectious virus within nasal cavity, submandibular lymph nodes, and the spleen in those animals challenged with CB MPXV suggesting initial replication within the primary site of infection (nasal cavity) followed closely by lymphatic spread. Although viral DNA was detected in the blood after that of the spleen and submandibular lymph node, it is possible that a primary, transient viremia occurred but was not captured. The temporal similarity of viral detection in nasal cavity, submandibular lymph nodes, tonsils, spleen, and liver, within the WA MXPV infected animals, supports the supposition that virus initially spread simultaneously through lymphatics (lymph nodes and tonsils) and blood stream (spleen and liver).

As seen within this and other MPXV animal models, detection of antibodies for all animals occurred at the same time or shortly after cutaneous lesion presentation [26]. However, sera from 2 animals were able to neutralize on day 9, before lesion onset. Most likely this neutralization is due to IgM antibodies, which previous data has suggested our OPXV ELISA assay does not adequately capture (unlike IgG antibodies). Therefore we are likely missing the initial rise in immunoglobulins.

Neutrophils were prominent within the red pulp of the spleen early in infection with both MPXV clades. Pathological changes were similar between the clades as the study progressed. Day 9 changes included dermal lesions characterized by epidermal vacuolation and inflammation along with varying lymphoid tissue necrosis. Additionally, for CB MPXV infected animals, splenic necrosis was observed. Day 12 animals had more appreciable pathological changes including multifocal necrosis in the oral and pharyngeal mucosal epithelia, liver, nasal cavity, uterus, spleen, small intestines, lymph nodes, and uterus. By study end, dermal fibrosis, hyperkeratosis, and crusting were the only findings. Comparing 2 animals (1 animal from each MPXV clade) that succumbed to disease on day 12, both animals demonstrated abundant viral antigen in all organs except the brain.

In the current study we were able to show that when cells are undergoing apoptosis, they generally colocalize with the virus suggesting that the viral invasion of these cells triggers the apoptotic response. Based on our study, there is suggestion that CB MXPV causes more apoptosis within the spleen compared to WA MPXV. Our results are in contrast to* in vitro* results which showed that WA MPXV caused increased apoptosis compared to CB MPXV via fluorescence-activated cell sorting analysis of AnnexinV staining [27]. This could be due to the fact that* in vitro* results are not adequately showing what is occurring* in vivo* or something specifically different within this animal model. Additional* in vivo* studies need to be performed in order to determine the cause of this discrepancy. Another interesting observation was that while there were a large number of cells labeled for apoptosis within the spleen, the liver had only a minimal trend of increased apoptotic cells, even though these 2 tissues had similarly high loads of viral burden in infected animals. Either the cells within the spleen are better equipped to initiate cell death when viral invasion occurs or the virus is more efficiently able to suppress apoptosis within the liver compared to the spleen; or perhaps a combination of these possibilities is the answer. Alternatively the virus may be preferentially initiating apoptosis within the spleen in order to release virions from within the cells to enhance viral spread from this lymphatic organ. Apoptosis is a complex signaling pathway that can be triggered by different stimuli. There are 2 main pathways that result in apoptosis, the intrinsic (mitochondrial) and extrinsic (death receptor) pathways; it is possible for both the intrinsic and extrinsic pathways to be activated simultaneously within tissues [28]. Interestingly, it has been reported that activation of Kupffer cells within the liver leads to secretion of the cytokine tumor necrosis factor (TNF) thereby activating the extrinsic apoptotic pathway [29–31]. Kupffer cells also express the Fas ligand which can also lead to apoptosis within hepatocytes [31]. As poxvirus proteins have been shown to inhibit both TNF and Fas receptor-induced apoptosis [32], this could explain why we did not see a dramatic increase in apoptosis within the liver as was seen in the spleen; the virus may be circumventing the host induced apoptotic response within the liver through inhibition of these specialized macrophages, Kupffer cells. The activation of apoptosis pathway(s) within different tissues as well as inhibition of these pathways in response to MPXV infection should be investigated as this may help to explain the differences in MPXV clade pathogenicity. Additional* in vivo* studies with more samples would be useful in elucidating the differences we observed.

**Figure 10:**
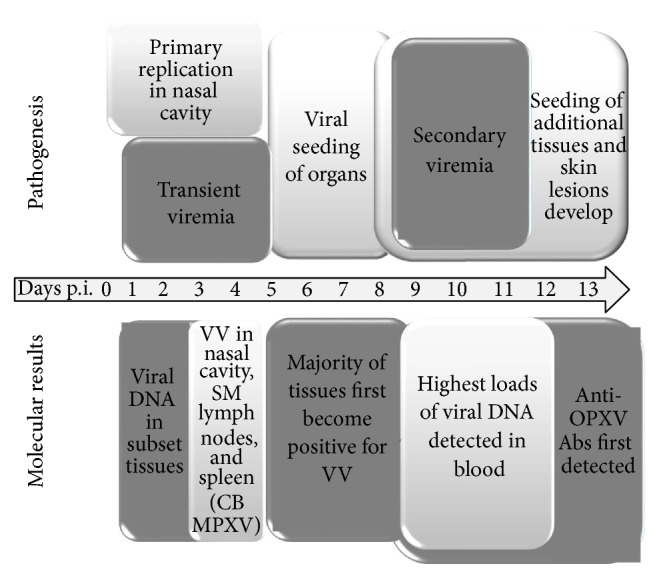
Proposed model for MPXV pathogenesis within prairie dogs. Time after infection is shown in days; VV: viable virus; SM: submandibular; CB: Congo Basin; Abs: antibodies.

Although our understanding of MPXV pathogenesis* in vivo* continues to increase, there is still much to learn. In early studies, a model based on MPXV challenge of cynomolgus monkeys was used to describe the viral pathogenesis after infection. The investigators hypothesized that, after a primary route of infection (intramuscular inoculation mimicking a bite from an infected animal), virus multiplies at this primary site leading to virus multiplication occurring simultaneously in the lymphatics and blood stream (primary viremia) followed by the draining lymph nodes, spleen, and tonsils [33]. This is followed by virus release into systemic lymph nodes and a secondary viremia. The secondary viremia results in skin and other organs infection leading to clinical signs of disease including cutaneous lesion presentation. Our studies utilizing intranasal inoculation to mimic respiratory exposure suggest a similar time course within the prairie dog MPXV model. Our data suggests that the virus replicates at the primary site of infection (nasal cavity) followed closely by regional lymphatic tissues. This results in a transient viremia leading to viral spread and replication in target organs followed by a secondary viremia leading to characteristic disseminated cutaneous lesions. Our proposed pathogenesis model for MPXV within the prairie dog has been depicted within Figure 10. These findings allow for the better understanding of the pathogenesis of MPXV within a relevant animal model, including identifying sites that are important during early viral replication and cellular response to viral infection. This data could prove to be useful in understanding the disease differences observed between the MPXV clades and understanding the risk of viral transmissions and development of therapeutic and biologic agents that could halt transmission between hosts.

Although we have shown with this and previous studies utilizing the prairie dog MXPV model that there are indeed differences in the pathogenicity between the two MPXV clades (similar to that seen during human MPXV infections), we have not determined the cause of these differences. CB MPXV causes increased morbidity and mortality, as well as a higher rate of transmission within humans as well as in our animal model. During a CB MPXV outbreak in Africa, up to 6 sequential interhuman transmission events have been documented [6]. In comparison, WA MPXV has never been associated with person to person transmission as the sole mode of transmission [7, 8]. Utilizing the prairie dog MPXV model to compare respiratory transmission of the MPXV clades, we housed infected animals 4 inches apart from naïve animals and found that none of the WA MPXV infected animals transmitted to naïve animals; however 16.7% of naïve animals housed across from CB infected animals subsequently became infected [12]. We have shown through the current and previous studies that CB MPXV infected prairie dogs consistently shed slightly larger loads of viable virus compared to WA MXPV. Additionally, through the current study, we see that CB MXPV infected animals have a slightly earlier viral kinetics timeline; however WA MXPV infected prairie dogs seem to shed virus for a slightly longer period of time. Although we have not yet identified the cause(s) of the differences in MPXV clade pathogenicity, we consistently show that, through utilization of the prairie dog MPXV model, we may yet understand why these genetically similar viruses result in different disease presentation and pathogenicity within humans.

## Supplementary Material

The mean percent value change from day zero to necropsy for each blood chemistry was compared between WA MPXV infected animals, CB MPXV infected animals and PBS controls, controlling for day of necropsy in Supplementary Table 1. The value change from day zero to necropsy for each blood chemistry was compared between groups and statistically analzyed.
Paraffin-embedded prairie dog liver and spleen sections were stained with the Millipore Apoptosis kit and apoptotic cells were counted. Counting of positive cells was performed by randomly moving each slide until 10 adjacent fields were counted; average positive cells ± the standard deviation is shown. Apoptotic cells were identified by a red-brown staining nucleus and compared between groups.
Mean viral loads (pfu/gram of tissue) were determined in tissues from prairie dogs intranasally challenged (8x10^3pfu) with West African (WA) MPXV in supplementary figure 1. Samples were evaluated for the presence of virus and were grouped by initial detection of viable virus (day 6, 9 or 12 p.i.)
Mean viral loads (pfu/gram of tissue) were determined in tissues from prairie dogs intranasally challenged (8x10^3pfu) with Congo Basin (CB) MPXV in supplementary figure 2. Samples were evaluated for the presence of virus and were grouped by initial detection of viable virus (day 4, 6, 9 or 12 p.i.)

## Figures and Tables

**Table 1:** Clinical and laboratory findings in prairie dogs intranasally challenged (8 × 10^3^ pfu) with West African (WA) MPXV.

Sacrifice day p.i.	Prairie dog #	Max. # of cutaneous lesions observed	OPXV antibodies detected	Neutralizing antibodies detected	Clinical symptoms	Gross pathology	Poxvirus IHC	Viable virus (#positive/#tested)
2	PD9	0	Neg.	Neg.	N.O.	N.O.	N.E.	0/28
	PD10	0	Neg.	Neg.	N.O.	N.O.	N.E.	0/28

4	PD11	0	Neg.	Neg.	N.O.	N.O.	N.O.	0/28
	PD12	0	Neg.	Neg.	N.O.	N.O.	N.O.	0/28

6	PD13	0	Neg.	Neg.	N.O.	N.O.	N.O.	10/28
	PD14	0	Neg.	Neg.	N.O.	N.O.	N.O.	10/28

9	PD15	0	Neg.	+	R.D.	Pale kidneys	LN, epithelial, dermis	15/28
	PD16	0	Neg.	Neg.	R.D.	N.O.	LN, spleen	16/28

12	PD17	20	+	N.E.	Inappetence	Lymphadenopathy, inflamed oviducts	Buccal/caudal pharyngeal mucosa, uterus, mesenchymal cells (mononuclear inflammatory cells/reactive fibroblasts)	21/28
	PD18	12	+	N.E.	Inappetence	Lymphadenopathy	Epithelial, tonsil, fibroblasts	10/28
	PD21^†^	3	N.E.	N.E.	Inappetence, nasal discharge	Hemorrhage of uterine horns and adipose tissue associated with the round ligament, hyperemic areas associated with the ovaries and inflamed oviducts, and hepatomegaly	All tissues positive for antigen except for brain	26/28

17	PD19	>20	+	+	Inappetence	Lymphadenopathy, hemorrhagic foci in the adipose tissue associated with lymph nodes and multiple, mild foci of serosal hemorrhage on the lungs	Peritesticular adipose tissue	14/28

24	PD20	5	+	N.E.	N.O.	N.O.	N.E.	0/28
	PD22	20	+	N.E.	N.O.	Pericarditis	N.E.	1/28

Groups of prairie dogs were serially sacrificed (days 2, 4, 6, 9, 12, 17, and 24) following challenge (*n* = 14). Animals were observed for clinical symptoms, antibody production, and lesion presentation. Gross observations were recorded and postmortem samples were evaluated for presence of virus and histopathological changes. R.D.: respiratory depression under anesthesia; N.O.: nothing observed; N.E. not examined.

†: unscheduled death.

**Table 2:** Clinical and laboratory findings in prairie dogs intranasally challenged (8 × 10^3^ pfu) with Congo Basin (CB) MPXV.

Sacrifice day p.i.	Prairie dog number	Maximum number of lesions observed^◊^	OPXV antibodies detected	Neutralizing antibodies detected	Clinical symptoms	Gross pathology	Poxvirus IHC	Viable virus (#positive/#tested)
2	PD23	0	Neg.	Neg.	N.O.	N.O.	N.E.	0/28
	PD24	0	Neg.	Neg.	N.O.	N.O.	N.E.	0/28

4	PD25	0	Neg.	Neg.	N.O.	Foci of serosal congestion in stomach and colon	N.O.	2/28
	PD26	0	Neg.	Neg.	N.O.	Foci of serosal congestion in stomach and lung	N.O.	2/28

6	PD27	0	Neg.	Neg.	N.O.	N.O.	N.O.	8/28
	PD28	0	Neg.	Neg.	N.O.	N.O.	N.O.	12/28

9	PD29	0	Neg.	+	N.O.	N.O.	Small intestine	16/28
	PD30	17	Neg.	Neg.	R.D., nasal discharge, died while under anesthesia	Multiple, randomly distributed foci of parenchymal hemorrhage on lungs	Small intestine, trachea, epithelial staining (unknown location)	25/28

12/13	PD31	5	+	N.E.	Facial edema	N.O.	Epiglottis associated with necrotic lesion, tongue, small intestine	19/28
	PD32^†^	4	N.E.	N.E.	R.D., inappetence, facial edema	Multifocal hemorrhagic lesions in the jejunum and lymphadenopathy/necrotic submandibular lymph nodes	All tissues positive for antigen except for brain	28/28
	PD36^†^	14	+	N.E.	Nasal discharge, inappetence, periocular erythema and epiphora, distended abdomen	Gastrointestinal distention uterus had a focal serosal hemorrhage, multifocal hemorrhagic lesions in the periovarian adipose tissue, hemorrhagic MALT in the peritoneum, mucosal hemorrhage in stomach	Rostral nasal cavity, epidermal with some dermal mesenchymal cells	24/28

17	PD33	>20	+	N.E.	Inappetence, facial edema, bloody nose/mouth, nasal discharge	Foci of hemorrhage in the jejunum and multifocal hemorrhagic lesions in the periovarian adipose tissue	Mesenchymal cell staining in mid dermis	11/28
	PD34	6	+	+	Facial edema, nasal discharge	N.O.	N.E.	3/28

24	PD35	13	+	N.E.	Inappetence	N.O.	N.E.	0/28

Groups of prairie dogs were serially sacrificed (days 2, 4, 6, 9, 12, 17, and 24) following challenge (*n* = 14). Animals were observed for clinical symptoms, antibody production, and lesion presentation. Gross observations were recorded and postmortem samples were evaluated for presence of virus and histopathological changes. R.D.: Respiratory depression while under anesthesia; N.O.: nothing observed; N.E.: not examined.

◊: maximum number of lesions at any time-point throughout study, not restrictive to day of necropsy.

†: unscheduled death.

**Table 3:** Viral kinetics and tissue tropism of MPXV infection within the prairie dog.

Tissue sample	West Africa MPXV			Congo Basin MPXV		
	DNA presence (days)	Virus presence (days)	Peak mean viral load value and day	DNA presence (days)	Virus presence (days)	Peak mean viral load value and day
Nasal cavity	4–24	6–17	7.7 × 10^8^ Day 9	2–17	4–17	2.6 × 10^8^ Days 12/13

Salivary glands/SM lymph nodes	2–24	6–12	6.8 × 10^5^ Day 6	4–24	4–13	9.9 × 10^7^ Day 6

Spleen	4–24	6–12	4.9 × 10^8^ Day 12	4–17	4–13	7.5 × 10^8^ Days 12/13

Tongue	4–24	6–17	2.1 × 10^7^ Day 12	4–17	6–17	4.8 × 10^7^ Days 12/13

Tonsils	4–24	6–12	7.7 × 10^7^ Day 12	4–17	6–13	4.6 × 10^6^ Days 12/13

Oropharynx	4–24	6–17	4.1 × 10^6^ Day 12	2–24	6–17	2 × 10^7^ Days 12/13

Inner cheek	6–24	6–17	2.4 × 10^7^ Day 12	4–17	6–17	2.8 × 10^7^ Days 12/13

Trachea	6–24	6–12	2.7 × 10^6^ Day 12	4–24	6–13	1 × 10^7^ Day 9

Lungs	4–17	6–12	5.9 × 10^7^ Day 12	4–24	6–13	3.9 × 10^7^ Days 12/13

Liver	4–17	6–12	5.7 × 10^8^ Day 12	2–17	6–12	2.9 × 10^9^ Days 12/13

Ileum	4–24	6–12	2.4 × 10^7^ Day 12	6–24	6–13	4.2 × 10^7^ Days 12/13

Duodenum	6–24	6–9	6.8 × 10^3^ Day 9	4–24	6–13	3.9 × 10^6^ Days 12/13

Jejunum	6–17	6–12	1.1 × 10^7^ Day 12	9–17	6–13	3.9 × 10^7^ Days 12/13

Eyelid	6–24	9–17	9.7 × 10^7^ Day 17	2–17	6–17	1.9 × 10^7^ Days 12/13

Eye	6–24	9–17	1.2 × 10^7^ Day 12	4–17	6–13	6.2 × 10^6^ Days 12/13

Kidney	6–24	9–12	3.8 × 10^6^ Day 12	6–17	9–13	4 × 10^6^ Days 12/13

Gonad	6–24	9–17	7.6 × 10^7^ Day 12	4–24	6–17	1 × 10^7^ Days 12/13

Belly skin	6–24	9–17	3.3 × 10^5^ Day 12	4–24	9–17	3.9 × 10^6^ Days 12/13

Mes. lymph node	4–24	12	1.4 × 10^7^ Day 12	6–17	9–13	1.2 × 10^6^ Days 12/13

Stomach	4–24	12	4.3 × 10^5^ Day 12	4–24	9–13	3.1 × 10^6^ Days 12/13

Brain	6–24	12–17	7.1 × 10^4^ Day 12	6–17	9–13	1.9 × 10^5^ Days 12/13

Heart	4–24	12	1.4 × 10^6^ Day 12	4–17	9–13	7.6 × 10^6^ Days 12/13

Pancreas	6–17	12	2.8 × 10^6^ Day 12	4–17	6–13	3.9 × 10^7^ Days 12/13

Urine/bladder	6–24	12–17	3.5 × 10^6^ Day 12	9–24	9–13	4.9 × 10^6^ Days 12/13

Gallbladder	6–24	12–17	3.8 × 10^4^ Day 12	4–24	12-13	2.2 × 10^6^ Days 12/13

Feces	12–24	12–24	6.5 × 10^6^ Day 12	12–24	12–17	1.1 × 10^7^ Days 12/13

Lesion	12–24	12–24	7.8 × 10^7^ Day 17	12–24	12–17	2.3 × 10^8^ Days 12/13

Prairie dogs were intranasally challenged (8 × 10^3^ p.f.u.) with West African (WA; *n* = 14) or Congo Basin (CB; *n* = 14) MPXV. Groups of prairie dogs were serially sacrificed (days 2, 4, 6, 9, 12, 17, and 24) following challenge. Postmortem samples were evaluated for presence of virus by real-time PCR and tissue culture. The time course of viral shedding for each tissue (viral DNA and viable virus) is shown above as is the peak viral load and day p.i. that the peak viral load was seen for each tissue. Peak viral loads were compared between MPXV clades; *p* values for these comparisons were not statistically significant when comparing each tissue type (CB versus WA) and controlling for day of necropsy (*p* value range of 0.21–1).

## References

[1] Reynolds M. G., Carroll D. S., Karem K. L. (2012). Factors affecting the likelihood of monkeypox's emergence and spread in the post-smallpox era. *Current Opinion in Virology*.

[2] Hutson C. L., Lee K. N., Abel J. (2007). Monkeypox zoonotic associations: Insights from laboratory evaluation of animals associated with the multi-state US outbreak. *American Journal of Tropical Medicine and Hygiene*.

[3] Reed K. D., Melski J. W., Graham M. B. (2004). The detection of monkeypox in humans in the Western Hemisphere. *The New England Journal of Medicine*.

[4] Chen N., Li G., Liszewski M. K. (2005). Virulence differences between monkeypox virus isolates from West Africa and the Congo basin. *Virology*.

[5] Likos A. M., Sammons S. A., Olson V. A. (2005). A tale of two clades: monkeypox viruses. *Journal of General Virology*.

[6] Learned L. A., Reynolds M. G., Wassa D. W. (2005). Extended interhuman transmission of monkeypox in a hospital community in the Republic of the Congo, 2003. *The American Journal of Tropical Medicine and Hygiene*.

[7] Breman J. G., Ruti K., Steniowski M. V. (1980). Human monkeypox, 1970–79. *Bulletin of the World Health Organization*.

[8] Foster S. O., Brink E. W., Hutchins D. L. (1972). Human monkeypox. *Bulletin of the World Health Organization*.

[9] Henderson D. A. (2002). Smallpox: clinical and epidemiologic features. *Medicine and Health, Rhode Island*.

[10] Hutson C. L., Olson V. A., Carroll D. D. (2009). A prairie dog animal model of systemic orthopoxvirus disease using west African and Congo Basin strains of Monkeypox virus. *Journal of General Virology*.

[11] Hutson C. L., Carroll D. S., Self J. (2010). Dosage comparison of Congo Basin and West African strains of monkeypox virus using a prairie dog animal model of systemic orthopoxvirus disease. *Virology*.

[12] Hutson C. L., Gallardo-Romero N., Carroll D. S. (2013). Transmissibility of the monkeypox virus clades via respiratory transmission: investigation using the prairie dog-monkeypox virus challenge system. *PLoS ONE*.

[13] Hutson C. L., Carroll D. S., Gallardo-Romero N. (2011). Monkeypox disease transmission in an experimental setting: prairie dog animal model. *PLoS ONE*.

[14] Keckler M. S., Carroll D. S., Gallardo-Romero N. F. (2011). Establishment of the black-tailed prairie dog (*Cynomys ludovicianus*) as a novel animal model for comparing smallpox vaccines administered preexposure in both high- and low-dose monkeypox virus challenges. *Journal of Virology*.

[15] Smith S. K., Self J., Weiss S. (2011). Effective antiviral treatment of systemic orthopoxvirus disease: ST-246 treatment of prairie dogs infected with monkeypox virus. *Journal of Virology*.

[16] Hudson P. N., Self J., Weiss S. (2012). Elucidating the role of the complement control protein in monkeypox pathogenicity. *PLoS ONE*.

[17] Li Y., Olson V. A., Laue T., Laker M. T., Damon I. K. (2006). Detection of monkeypox virus with real-time PCR assays. *Journal of Clinical Virology*.

[18] Karem K. L., Reynolds M., Braden Z. (2005). Characterization of acute-phase humoral immunity to monkeypox: use of immunoglobulin m enzyme-linked immunosorbent assay for detection of monkeypox infection during the 2003 north American outbreak. *Clinical and Diagnostic Laboratory Immunology*.

[19] Guarner J., Johnson B. J., Paddock C. D. (2004). Monkeypox transmission and pathogenesis in prairie dogs. *Emerging Infectious Diseases*.

[20] Lehmann E. L. (1975). *Nonparametrics: Statistical Methods Based on Ranks*.

[21] Sogayar M. I., Yoshida E. L. (1995). Giardia survey in live-trapped small domestic and wild mammals in four regions in the southwest region of the state of São Paulo, Brazil. *Memórias do Instituto Oswaldo Cruz*.

[22] Marino M. R., Brown T. J., Waddington D. C., Brockie R. E., Kelly P. J. (1992). *Giardia intestinalis* in North Island possums, house mice and ship rats. *New Zealand Veterinary Journal*.

[23] Pinter A. J., O'Dell W. D., Watkins R. A. (1988). Intestinal parasites of small mammals from Grand Teton National Park. *The Journal of Parasitology*.

[24] Medina-Esparza L., Macías L., Ramos-Parra M., Morales-Salinas E., Quezada T., Cruz-Vázquez C. (2013). Frequency of infection by neospora caninum in wild rodents associated with dairy farms in aguascalientes, Mexico. *Veterinary Parasitology*.

[25] Ferroglio E., Pasino M., Romano A., Grande D., Pregel P., Trisciuoglio A. (2007). Evidence of Neospora caninum DNA in wild rodents. *Veterinary Parasitology*.

[26] Wenner H. A., Kamitsuka P., Macasaet F., Kidd P. (1967). Pathogenesis of monkey pox. *Antimicrobial Agents and Chemotherapy*.

[27] Kindrachuk J., Arsenault R., Kusalik A. (2012). Systems kinomics demonstrates Congo Basin monkeypox virus infection selectively modulates host cell signaling responses as compared to West African monkeypox virus. *Molecular & Cellular Proteomics*.

[28] Strasser A., O'Connor L., Dixit V. M. (2000). Apoptosis signaling. *Annual Review of Biochemistry*.

[29] Kresse M., Latta M., Künstle G. (2005). Kupffer cell-expressed membrane-bound TNF mediates melphalan hepatotoxicity via activation of both TNF receptors. *Journal of Immunology*.

[30] Malhi H., Guicciardi M. E., Gores G. J. (2010). Hepatocyte death: a clear and present danger. *Physiological Reviews*.

[31] Canbay A., Feldstein A. E., Higuchi H. (2003). Kupffer cell engulfment of apoptotic bodies stimulates death ligand and cytokine expression. *Hepatology*.

[32] Bertin J., Armstrong R. C., Ottilie S. (1997). Death effector domain-containing herpesvirus and poxvirus proteins inhibit both Fas- and TNFR1-induced apoptosis. *Proceedings of the National Academy of Sciences of the United States of America*.

[33] Cho C. T., Wenner H. A. (1973). Monkeypox virus. *Bacteriological reviews*.

