# Supplementary material for: Comparison of Monkeypox Virus Clade Kinetics and Pathology within the Prairie Dog Animal Model Using a Serial Sacrifice Study Design
Source: Biomed Res Int. 2015 Aug 24;2015:965710. doi: 10.1155/2015/965710 (PMC4561332; doi:10.1155/2015/965710)
Supplement: Supplementary file 1 — The mean percent value change from day zero to necropsy for each blood chemistry was compared between WA MPXV infected animals, CB MPXV infected animals and PBS controls, controlling for day of necropsy in Supplementary Table 1. The value change from day zero to necropsy for each blood chemistry was compared between groups and statistically analzyed. Paraffin-embedded prairie dog liver and spleen sections were stained with the Millipore Apoptosis kit and apoptotic cells were counted. Counting of positive cells was performed by randomly moving each slide until 10 adjacent fields were counted; average positive cells ± the standard deviation is shown. Apoptotic cells were identified by a red-brown staining nucleus and compared between groups. Mean viral loads (pfu/gram of tissue) were determined in tissues from prairie dogs intranasally challenged (8x10^3pfu) with West African (WA) MPXV in supplementary figure 1. Samples were evaluated for the presence of virus and were grouped by initial detection of viable virus (day 6, 9 or 12 p.i.) Mean viral loads (pfu/gram of tissue) were determined in tissues from prairie dogs intranasally challenged (8x10^3pfu) with Congo Basin (CB) MPXV in supplementary figure 2. Samples were evaluated for the presence of virus and were grouped by initial detection of viable virus (day 4, 6, 9 or 12 p.i.) [file 965710.f1.pdf]

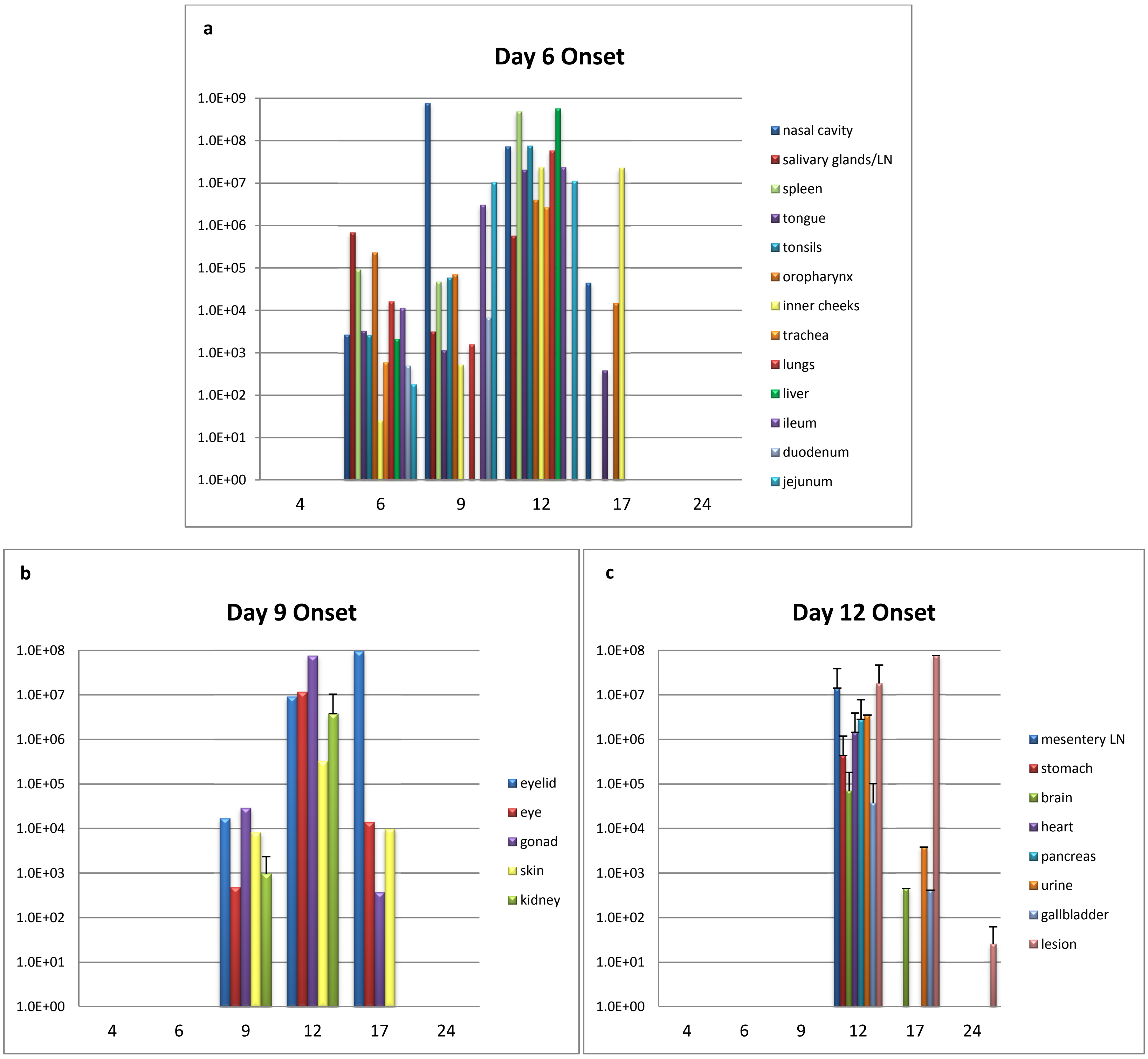

Supplementary Figure 1: Mean viral loads in tissues from prairie dogs intranasally challenged (8x10^3pfu) with West African (WA) MPXV. Groups of prairie dogs were serially sacrificed (days 2, 4, 6, 9, 12, 17 and 24) following challenge. Samples were evaluated for the presence of virus and were grouped by initial detection of viable virus (day 6 (a), 9 (b), or 12 (c) p.i.). pfu/gram of tissue are shown on a log scale.

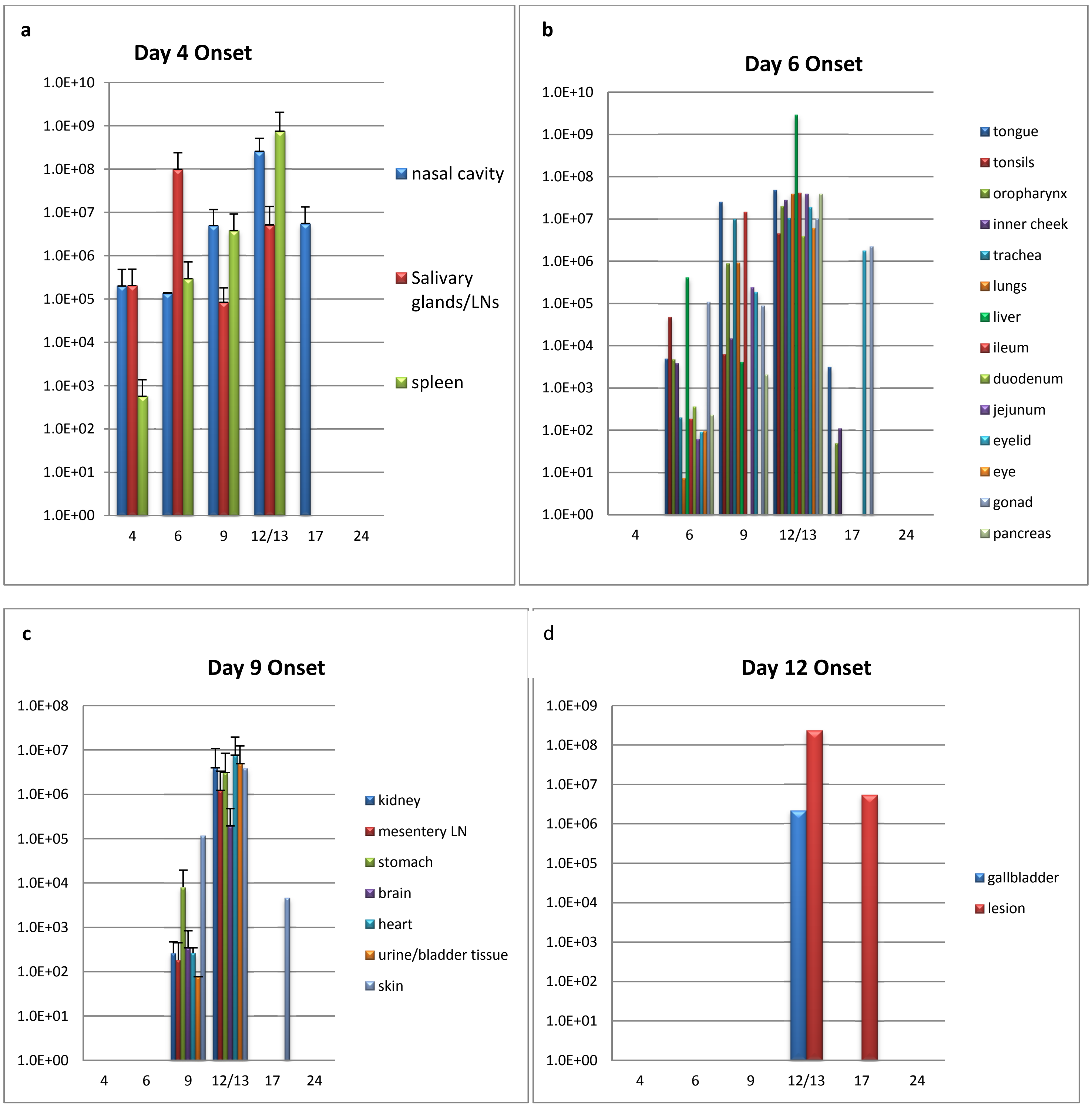

Supplementary Figure 2: Mean viral loads in tissues from prairie dogs intranasally challenged (8x10^3pfu) with Congo Basin (CB) MPXV. Groups of prairie dogs were serially sacrificed (days 2, 4, 6, 9, 12, 17 and 24) following challenge. Samples were evaluated for the presence of virus and were grouped by initial detection of viable virus (day 4 (a), 6 (b), 9 (c) or 12 (d) p.i.). pfu/gram of tissue are shown on a log scale.

	(A) West African MPXV							(B) Congo Basin MPXV							(C) PBS						
	Necropsy day							Necropsy day							Necropsy day						
	2	4	6	9	12	17	24	2	4	6	9	12	17	24	2	4	6	9	12	17	24
NA	4.9%	3.7%	2.1%	-1.4%	1.4%	-2.16%	n/a	7.23%	2.98%	-8.66%	0%	-0.72%	-2.57%	-13.89%	n/a	n/a	6.62%	2.21%	n/a	0%	n/a
K	63.9%	-1.7%	8.7%	-28.2%	3.66%	1.71%	n/a	15.8%	30.87%	-42.86%	42.7%	1.85%	2.17%	-10.7%	n/a	n/a	14.89%	35.42%	n/a	8.70%	n/a
tCO2	1.79%	-9.2%	n/a	6.06%	n/a	-1.2%	n/a	36.3%	8.14%	29.63%	0.21%	-18.75%	-6.16%	0%	n/a	n/a	-5.88%	12.9%	n/a	n/a	n/a
CL	-3.56%	8.77%	n/a	3.75%	n/a	2.5%	n/a	0%	8.77%	n/a	6.17%	n/a	8.77%	n/a	n/a	n/a	7.59%	3.66%	n/a	n/a	n/a
GLU	-76%	36%	49.6%	16.35%	5.9%	69.58%	n/a	-50.93%	65.63%	14.63%	12.09%	20.15%	24.48%	37.2%	n/a	n/a	49.15%	-22.64%	n/a	-3.74%	n/a
CA	10.05%	-5.23%	-0.94%	30.67%	-24.52%	-3.8%	n/a	30.42%	28.9%	45.92%	-3.73%	-5.94%	-12.9%	-13.4%	n/a	n/a	0%	-3.13%	n/a	-15.84%	n/a
BUN	11.16%	-2.5%	-36.8%	22.22%	10.55%	-17.7%	n/a	3.11%	10%	-25%	-4.55%	5.56%	-4%	50%	n/a	n/a	15%	-11.11%	n/a	8.70%	n/a
CRE	12.5%	6.1%	-25%	-22%	-21.4%	-23.6%	n/a	-4.44%	18.1%	-37.5%	22.22%	12.5%	0%	-55.56%	n/a	n/a	28.57%	28.57%	n/a	-22.22%	n/a
ALP	26.6%	8.84%	11.19%	-29.11%	3.03%	-36.9%	n/a	17.82%	13.76%	7.69%	-6.52%	-7.69%	-39.22%	-49.06%	n/a	n/a	77.78%	22.73%	n/a	-1.89%	n/a
ALT	-29.4%	-25.8%	125%	-30%	30%	-20%	n/a	-20.78%	15.18%	12.78%	35.71%	n/a	-29.76%	n/a	n/a	n/a	-8.33%	75%	n/a	-22.22%	n/a
AST	52.49%	-75.97%	n/a	-79.7%	n/a	-24.2%	n/a	26.99%	35.36%	-21.43%	35.36%	-62.75%	-5.78%	-8.70%	n/a	n/a	0%	-25%	n/a	n/a	n/a
TBIL	8.3%	25%	-33.3%	0	0	0	n/a	35.36%	8.33%	-16.67%	-16.67%	50	70.71%	0%	n/a	n/a	0%	0%	n/a	0%	n/a
ALB	-4.7%	-12.88%	68.4%	-9.09%	38.7%	-29.5%	n/a	-11.52%	-2.5%	18.32%	-10.44%	-22.27%	-61.22%	-30	n/a	n/a	-17.39%	-4.76%	n/a	52.4%	n/a
TP	-3.14%	-8.23%	-4.84%	-1.64%	-5.54%	8.02%	n/a	-8.93%	-2.4%	-12.48%	3.38%	-8.62%	-21.46%	-11.67%	n/a	n/a	-15.15%	-10.34%	n/a	-3.51%	n/a

Supplementary Table 1: Mean Percent Change: (Day zero value - Necropsy day value)/Day zero value in blood chemistry values for prairie dogs experimentally infected with West African MPXV (A), Congo Basin MPXV (B) or PBS control animals (C). The value change from day zero to necropsy for each blood chemistry was compared between groups. Wilcoxon rank-sum test p-values range from 0.1-1.0.

LIVER APOPTOSIS

PD#	Day p.i	Avg # of apoptotic cells	PD#	Day p.i	Avg # of apoptotic cells
PD2	PBS	0.9±0.7	PD2	PBS	0.9±0.7
**A.**	**WAMPXV**		**B.**	**CB MPXV**	
PD11	Day 4	1±0.6	PD25	Day 4	6.2±4.5
PD12	Day 4	0.7±0.6	PD26	Day 4	1.1±0.7
PD13	Day 6	4.4±2.0	PD27	Day 6	4.6±3.3
PD14	Day 6	5.3±2.9	PD28	Day 6	2.8±1.8
PD15	Day 9	10.1±11.0	PD29	Day 9	7.9±5.0
PD16	Day 9	0.8±1.0	PD30	Day 9	4.7±3.6
PD21	Day 12	6.9±4.0	PD32	Day 12	3.6±1.3

SPLEEN APOPTOSIS

PD#	Day p.i.	Avg # of apoptotic cells	PD#	Day p.i	Avg # of apoptotic cells
PD2	PBS	3.4±1.6	PD2	PBS	3.4±1.6
**C.**	**WA MPXV**		**D.**	**CB MPXV**	
PD11	Day 4	1.6±1.0	PD25	Day 4	1.5±1.5
PD12	Day 4	1.4±1.5	PD26	Day 4	2±1.9
PD13	Day 6	10.7±7.8	PD27	Day 6	17.2±10.0
PD14	Day 6	8.7±5.0	PD28	Day 6	12±7.2
PD15	Day 9	17.3±8.4	PD29	Day 9	22.1±8.7
PD16	Day 9	15±4.3	PD30	Day 9	34.9±13
PD21	127	Day 12	14.8±11.1	Day 12	TMTC (>100)

Supplementary Table 2 : Number of apoptotic cells in liver and spleen from MPXV infected prairie dogs. Prairie dogs intranasally
challenged (8x10^3pfu) with West African (WA; A, C) or Congo Basin (CB; B, D)MPXV were serially sacrificed (days 2, 4, 6, 9, 12, 17 and 24)

following challenge. Paraffin-embedded prairie dog liver (A, B) and spleen (C, D) sections were stained with Millipore Apoptosis kit and
apoptotic cells were counted. Counting of positive cellswas performed by randomly moving each slide until 10 adjacent fields were counted;
average positive cells ± the standard deviation is shown. Apoptotic cells were identified by red-brown staining nucleus. (immunoperoxidase method,
Vector NovaRed chromogen, hematoxylin counterstain).
